# Low cholesterol and risk of violence in forensic inpatients with schizophrenia, personality disorder or dual diagnosis: same or different?

**DOI:** 10.1192/j.eurpsy.2025.10051

**Published:** 2025-06-20

**Authors:** Piyal Sen, Mehr-un-Nisa Waheed, Fern Taylor, Rebecca Mottram, Quazi Haque, Alex Blakemore, Veena Kumari

**Affiliations:** 1 Centre for Cognitive and Clinical Neuroscience, College of Health, Medicine and Life Sciences, Brunel University of London, Uxbridge, UK; 2 Department of Psychology, College of Health, Medicine and Life Sciences, Brunel University of London, Uxbridge, UK; 3 Department of Psychosis Studies, Institute of Psychiatry, Psychology and Neuroscience, King’s College London, London, UK; 4 Elysium Healthcare, Borehamwood, Hertfordshire, UK; 5 Department of Life Sciences, College of Health, Medicine and Life Sciences, Brunel University of London, London, UK; 6 Department of Metabolism, Digestion and Reproduction, Imperial College London, London, UK; 7 College of Medicine, Nursing, and Health Science, University of Galway, Galway, Republic of Ireland

**Keywords:** lipids, risk assessment, schizophrenia, secure, statins

## Abstract

**Background:**

Violence and suicidality are common in forensic inpatients, most commonly with schizophrenia (SZ), personality disorder (PD), or comorbid SZ and PD (dual diagnosis, DD). There are no biological markers used in risk assessment tools. Lipids may provide a useful biomarker to aid violence prediction, but the roles of diagnosis and sex remain unclear. We therefore investigated lipids in adult forensic inpatients in association with the risk of violence and suicidality by primary diagnosis and sex.

**Method:**

Anonymized data were obtained for all eligible inpatients [*n* = 230; 114 SZ (75 males), 77 PD (40 males), 39 DD (20 males)] who had been admitted (2002–2021) to Elysium Healthcare (UK-wide) medium/low-secure facilities on lipids, age, sex, diagnosis, medication, risk of violence and suicidality, as well as days in seclusion and on high observations due to violence.

**Results:**

Mean total cholesterol (TC) in the patient sample (4.57, s.d. = 1.09) was lower, relative to the age- and sex-corrected UK population norm (4.91 mmol/l). PD (4.46 ± 1.08 mmol/l) and DD (4.24 ± 0.82 mmol/l), compared to SZ patients (4.77 ± 1.14 mmol/l), had significantly lower TC (not explained by statin use; no effect or interaction involving sex). Lower TC had significant though small associations with more days in seclusion or high observation levels due to violence across all patients, and marginally with suicidality in females.

**Conclusions:**

A low TC-violence (towards others) link exists not only for SZ but also for PD and DD and for males and females, encouraging further enquiry into lipids as a biomarker to aid violence prediction in secure care.

## Introduction

Most forensic inpatients are diagnosed with schizophrenia (SZ), personality disorder (PD), or have a dual diagnosis of both SZ and PD (DD) [1], a comorbidity that is considered to significantly increase the risk of violence [2]. According to some estimates [3], almost 1 in 5 patients with psychotic illnesses, mostly SZ, have a history of violence. Several meta-analytical studies and reviews [4–10] find that people with psychosis, even without substance misuse, have an increased risk of violence. This is true for community samples, forensic samples, and for males as well as females [11–18]. High rates of suicide and self-harm are also reported in SZ, with suicide mortality estimated to vary from 5 to 10% [19, 20] and the rates of suicide attempts ranging between 18 and 55% [21, 22]. The mechanism of the link between SZ and violence towards others, however, may be at least partially different from the mechanism of the link with suicide [23].

Regarding violence in PD [24, 25], there is a three to fourfold increased risk when compared to the general population [26, 27]. This risk is the highest in antisocial PD, with a recent systematic review finding a seven-fold increase [28]. There is also a strong connection between borderline PD and risk, including violent behavior, criminality, suicide, and self-harm [29]. Comorbid antisocial and borderline PD contributes to over-aggressive and criminalized behavior in prison and secure hospital samples [30–36], both for males and females [37].

Inpatient violence is also a significant issue, with one study reporting such violence to be present in approximately 48% of forensic psychiatric patients, with the highest rates in countries such as the UK and the US [38], leading to adverse consequences for staff [39]. A small subgroup of detained patients tends to be responsible for the largest proportion of violent incidents in hospitals [40]. Forensic mental health services are tasked with predicting the risk of future violence by individual patients towards themselves and others but there are few, if any, reliable and objective or numerical parameters of risk to introduce greater consistency into this process [41]. To assist clinical decision-making, the use of structured tools and instruments for violence risk assessment has increasingly been advocated in clinical practice. Structured professional judgment tools provide a framework including formulation and risk management. These are resource-intensive but still have limited predictive accuracy [42–44]. There are also doubts about their full applicability to women [45, 46]. A recent meta-analysis [47] found the evidence to be mixed for the current risk assessment tools used in forensic mental health. The predictive performance of such tools is worse than tools predicting imminent violence, which are better at separating high-risk and low-risk patients [48]. Studies also appear to indicate that modifiable clinical risk factors provide little advantage over static risk factors [42]. Therefore, efforts need to be directed at increasing the range of risk factors that could reliably separate high-risk from low-risk patients.

Currently, there are no biological tools that are included in any structured professional judgment instrument [41] but there is growing evidence that lipids may provide a useful biomarker to aid violence and suicidality prediction in forensic mental health patients. A systematic review [49] supported a link between low total cholesterol (TC) and violence to others in SZ, finding this association to be present in six of the nine studies (66.66%) that investigated this link. There was a relatively weaker link between low TC and suicidality (present in only 8 of 20 studies; 40%) [49]. Two studies [50, 51] also investigated and found a link between low high-density lipoprotein (HDL) and violence, but only for men and not women, and one study [52] found a link between lower low-density lipoprotein (LDL) and aggression. With regards to triglycerides (TG), only two of the five studies found a significant link, one between low TG and increased violence [52] and the other between high TG and increased violence [53]. Further to this systematic review [49], a recent study [54] in female SZ patients reported significantly lower HDL in aggressive patients, compared to non-aggressive patients; no significant differences were found for LDL, and no direct association between HDL and suicidality or aggression rating were found within the aggressive group. Further recent studies have found associations between appetitive aggression and low TG, as well as low TC, in male forensic SZ patients [55], and low HDL in people with psychotic disorders with a criminal history relative to those without such a history as well as healthy controls [56].

With respect to PD, lower TC levels have been identified to be a risk factor for self-mutilative and homicidal behavior in non-forensic PD samples [57–60]. Studies exploring this link in forensic PD inpatients [61, 62] have also demonstrated a low TC-violence link. Notably, all previous PD studies examined male-only samples and did not examine sex differences in TC-violence relationships despite potential differences in forensic profiles of males and females with PD [63]. Furthermore, no previous study has investigated lipids in relation to violence, self-harm and suicidality comprehensively in PD (or SZ) using multiple clinical parameters to assess risk, including ratings from structured professional risk assessment tools as well as information from restrictive interventions like seclusion and close observations to make a judgment on risk, or investigated this association specifically in patients with DD.

The main aims of the present study, therefore, were to examine: (i) the lipid profile, as indexed by the levels of TC, HDL, LDL and TG, of adult patients within secure services in relation to the population norms, and any differences based on diagnosis (SZ, PD, DD) and sex, and (ii) possible links between lipids and risk behaviors (violence, self-harm, and suicidal behaviors) across the entire patient sample, and within subgroups based on diagnosis and sex. We expected to find low lipid levels, especially TC, in our patient sample (since all would have a history of violence to others), compared to national lipid norms. We also expected to find a link between the risk of violence to others, self-harm and suicidality, and low lipid levels, especially TC.

## Method

### Study design and population

This study utilized data that had been gathered within the context of routine clinical care at medium and low secure units within Elysium Healthcare (comprising a network of 11 secure hospitals across the UK) and provided to the research team in an anonymized form solely for research purposes. The data were derived from Care Notes (an electronic patient record database) for all patients who had been admitted to and discharged between June 2002 and June 2021. Of the total initial pool of 870 patients, 230 met the diagnostic criteria for SZ, PD, or DD [64] and had their TC data available from a sample taken at, or around, admission to a medium/low secure facility.

The study was approved by the College of Health and Life Sciences Research Ethics Committee, Brunel University of London (23000-LR-May/2020-25478-2) and complies with the ethical standards of the relevant national and international committees on human experimentation and with the Helsinki Declaration of 1975, as revised in 2013.

### Data indices

For all patients, data on age, sex, body mass index (BMI), TC (and LDL, HDL, and TG where available) from routine blood tests, primary and secondary diagnoses, regular prescribed medication (antipsychotics, antidepressants, anxiolytics, mood stabilizers, antimuscarinic, and statins), risk of violence and length of stay were obtained. For patients with more than one blood tests, we calculated mean TC, LDL, HDL and TG scores given highly positive correlations (*p* < 0.001) between lipid levels at different time points.

Data on the risks of violence were available from a range of sources, including the violence risk assessment instrument Historical, Clinical and Risk HCR-20, version 3 [65] and the risk section of the Structured Assessment of Risk and Treatability (START) clinical assessment tool [66]. HCR-20 has 20 items, 10 in the History section (e.g., violence and antisocial behavior as a child, relationships, substance use), and five each in Clinical (e.g., violent ideation or intent, affective instability, treatment or supervision response) and Risk (e.g., living situation, personal support, stress or coping) sections, all scored on a 3-point Likert scale indicating prevalence, where 0 = “no,” 1 = “possibly” or “partially,” and 2 = “yes.” Only the risk section of the START which contains seven items (self-harm, suicide, unauthorized leave, substance abuse, self-neglect, being victimized, case specific risks) was considered, and each item was scored on a 3-point Likert scale to score the likelihood of the item being a risk for the individual, where 0 = “low,” 1 = “moderate,” and 2 = “high.” Other risk data extracted from case notes were the number of days spent in seclusion and the number of days spent on high levels of observation.

### Statistical analysis

All analyses were performed using the Statistical Package for Social Sciences (for Windows, version 28; IBM, New York, USA). Possible age differences were examined using a 3 (Diagnosis: SZ, PD, DD) x 2 (Sex: male, female) analysis of variance (ANOVA), and followed up with post-hoc mean comparisons. Given a significant Sex effect and Diagnosis x Sex interaction in age (see Results), TC, HDL, and TG data were analyzed using a 3 (Diagnosis) x 2 (Sex) analyses of covariance (ANCOVA), with age entered as a covariate. LDL data were not analyzed further due to missing data for >20% of the sample. Effect sizes, where reported, are partial eta squared (*ηp*^2^; the proportion of variance associated with a factor). Next, correlational analyses (Pearson’s *r* or Spearman *rho*) were used to examine possible links between lipids and risk behaviors (violence, self-harm, and suicidal behaviors) across all patients, and in subgroups based on primary diagnosis and sex. Alpha level for hypothesis testing was maintained at 0.05 in all analyses unless stated otherwise.

## Results

### Demographic and clinical variables

Of 230 patients, 114 patients had SZ (75 males, 39 females), 77 had PD (40 males, 37 females), and 39 had comorbid SZ and PD (20 males, 19 females) (Table 1). There was no significant main effect of Diagnosis in age but there was a significant Sex effect [*F* (1,224) = 18.15, *p* < 0.001; *np*^2^ = 0.075] with females being younger than males [mean years (s.d.): females, 31.83 (12.40); males, 38.85 (12.19]. There was also a significant Diagnosis x Sex interaction [*F* (2,224) = 3.56, *p* = 0.03; *np*^2^ = 0.031], explained by PD females being significantly younger than PD males (*p* < 0.001), and DD females being marginally younger than DD males (*p* = 0.05); SZ males and females were comparable in age (*p* = 0.24). Most patients were on some form of medication (Table 1).

**Table 1.** Sample characteristics

Demographics	Schizophrenia (SZ)			Personality disorder (PD)			Dual diagnosis (DD)		
Sex (%)	34.2% Female, 65.8% male			48.1% Female, 51.9% male			48.7% Female, 51.3% male		
Ethnicity (%)	76.3% White, 13.2% Black, 6.1% Asian, 4.4% Mixed			94.8% White, 1.3% Asian, 3.9% Mixed			94.9% White, 5.1% Black		
	Female (*n* = 39)	Male (*n* = 75)	All (*n* = 114)	Female (*n* = 37)	Male (*n* = 40)	All (*n* = 77)	Female (*n* = 19)	Male (*n* = 20)	All (*n* = 39)
	**Mean (s.d.)**	**Mean (s.d.)**	**Mean (s.d.)**	**Mean (s.d.)**	**Mean (s.d.)**	**Mean (s.d.)**	**Mean (s.d.)**	**Mean (s.d.)**	**Mean (s.d.)**
Age (years)	34.46 (12.48)	37.21 (11.30)	36.27 (11.72)	29.00 (11.85)	41.55 (14.15)	35.52 (14.46)	32.01 (12.78)	39.58 (10.67)	35.89 (12.21)
**Lipid profile**									
TC	4.97 (1.12)	4.66 (1.14)	4.77 (0.82)	4.55 (0.91)	4.37 (1.22)	4.46 (1.08)	4.11 (0.84)	4.35 (0.80)	4.23 (0.82)
HDL^a^	1.35 (0.55)	1.17 (0.48)	1.24 (0.51)	1.40 (0.58)	1.12 (0.48)	1.23 (0.55)	1.38 (0.56)	1.23 (0.57)	1.31 (0.57)
TG^b^	2.45 (1.53)	2.30 (1.19)	2.36 (1.31)	1.59 (0.63)	2.11 (1.07)	1.88 (0.93)	2.30 (0.83)	2.21 (0.86)	2.25 (0.83)
**Medication for psychiatric symptoms at admission**									
Antipsychotic (%)	85.1	62.7	70.2	51.4	32.5	40.3	78.9	100	89.7
Antidepressants (%)	35.9	13.3	21.1	29.7	17.5	23.4	47.4	20	33.3
Anxiolytics (%)	61.5	30.7	41.2	29.7	20	24.7	57.9	40	48.7
Mood Stabilizers (%)	20.5	26.7	24.6	10.8	5	7.8	36.8	45	41
Antimuscarinics (%)	5.1	0	1.8	0	0	0	15.8	5	10.3
Statins (%)	2.6	12	8.8	48.6	60	54.5	0	5	2.6
**TC in patients with and without statin**									
**TC**	Statin	No Statin	Statin		No Statin		Statin	No Statin	
	**Mean (s.d.)**	**Mean (s.d.)**	**Mean (s.d.)**		**Mean (s.d.)**		**Mean (s.d.)**	**Mean (s.d.)**	
	4.57 (0.87)	4.79 (1.16)	4.46 (0.99)		4.38 (1.18)		5.37 (0)	4.23 (0.78)	

a Data missing for 2 SZ females and 4 SZ males; 7 PD females and 6 PD males; and 1 DD female and 3 DD males.

b Data missing for 5 SZ females and 12 SZ males; 6 PD females and 3 PD males; and 6 DD female and 4 DD males.

Considering the current and historical risk profiles of the sample, females seemed to have a greater risk of historical violence (H1) and current violence (risk to others) than males across all groups and to have more historical traumatic experiences (H8), current victimization experiences (victimization, START), and judged to be currently at a higher risk of suicide (suicide, START). Females also generally had greater historical risk of violence linked to relationships and substance use across all diagnostic categories (Table 2).

**Table 2.** Clinical characteristics of the study groups (HCR 20, Y = Yes, N = No, P - Partial; START: L = Low, M = Medium, H = High)

		Schizophrenia (SZ)						Personality disorder (PD)						Dual diagnosis (DD)					
Assessment		Males (%)			Females (%)			Males (%)			Females (%)			Males (%)			Females (%)		
**HCR–20**		**Y**	**N**	**P**	**Y**	**N**	**P**	**Y**	**N**	**P**	**Y**	**N**	**P**	**Y**	**N**	**P**	**Y**	**N**	**P**
H1 (history of violence)	Child	22.4	55.1	22.4	18.5	59.3	22.2	33.3	57.1	9.5	17.2	51.7	31.0	68.4	15.8	15.8	58.8	35.3	5.9
	Adolescent	26.1	45.7	28.3	60.7	28.6	10.7	22.2	74.1	3.7	36.7	40.0	23.3	61.1	22.2	16.7	68.8	25.0	6.3
	Adult	52.5	45.9	1.6	79.4	20.6	0	44.4	51.9	3.7	53.3	46.7	0	89.5	10.5	0	100	0	0
H2 (other antisocial behavior)	Child	13.6	50.0	36.4	9.1	68.2	22.7	21.1	57.9	21.1	24.0	36.0	40.0	26.7	46.7	26.7	42.9	50.0	7.1
	Adolescent	36.5	40.4	23.1	53.6	21.4	25.0	30.4	60.9	8.7	36	32	32	52.9	23.5	23.5	68.8	12.5	18.8
	Adult	46.7	38.3	15.0	71.0	12.9	16.1	44.4	44.4	11.1	48.4	38.7	12.9	78.9	5.3	15.8	94.1	5.9	0
H3 (relationships)	Intimate	41.8	36.4	21.8	57.1	25.0	17.9	20.0	56.0	24.0	48.0	44.0	8.0	64.7	17.6	17.6	81.3	12.5	6.3
	Non-intimate	49.2	37.3	13.6	75.0	21.9	3.1	42.3	38.5	19.2	58.6	31.0	10.3	63.2	20.0	15.8	75.0	6.3	18.8
H4 (employment)		51.6	33.9	14.5	51.7	20.7	27.6	34.6	46.2	19.2	42.9	28.6	28.6	58.8	17.6	23.5	82.4	11.8	5.9
H5 (substance use)		45.5	51.5	3.0	61.1	30.6	8.3	37.9	48.3	13.8	43.8	28.1	28.1	78.9	10.5	10.5	88.2	11.8	0
H6 (major mental disorder)	Psychotic	58.1	41.9	0	71.4	25.7	2.9	37.0	44.4	18.5	51.6	35.5	12.9	68.4	15.8	15.8	94.1	5.9	0
	Mood	33.9	40.7	25.4	45.7	40.0	14.3	22.2	29.6	48.1	23.3	63.3	13.3	42.1	42.1	15.8	56.3	37.5	6.3
	Other	17.6	35.3	47.1	27.6	48.3	24.1	28.0	28.0	44.0	30.0	50.0	20.0	27.8	61.1	11.1	21.4	64.3	14.3
H7 (personality disorder)	Antisocial	31.3	40.6	28.1	23.1	50.0	26.9	40.0	40.0	20.0	35.7	35.7	28.6	44.4	16.7	38.9	46.2	15.4	38.5
	Other	32.3	19.4	48.4	50.0	23.1	26.9	34.8	43.5	21.7	37.9	51.7	10.3	52.6	26.3	21.1	75.0	8.3	16.7
H8 (traumatic experience)	Victim	45.5	32.7	21.8	61.3	22.6	16.1	32.0	52.0	16.0	43.3	46.7	10.0	73.7	5.3	21.1	75.0	6.3	18.8
	Adverse	39.7	31.0	29.3	62.5	21.9	15.6	29.6	63.0	7.4	39.3	39.3	21.4	66.7	6.7	26.7	76.5	11.8	11.8
H9 (violent attitudes)		45.9	34.4	19.7	51.5	21.2	27.3	44.4	29.6	25.9	53.3	33.3	13.3	70.0	5.0	15.0	77.8	5.6	16.7
H10 (treatment or supervision response)		50.8	41.5	7.7	65.7	25.7	8.6	48.1	40.7	11.1	41.9	45.2	12.9	73.7	15.8	10.5	100	0	0
**START**		**L**	**M**	**H**	**L**	**M**	**H**	**L**	**M**	**H**	**L**	**M**	**H**	**L**	**M**	**H**	**L**	**M**	**H**
Risk to Self		35.1	43.2	21.6	31.9	51.4	16.7	37.5	37.5	25.0	44.0	32.0	24.0	73.7	26.3	0	25.0	31.3	43.8
Risk to Others		83.3	12.5	4.2	67.6	21.6	10.8	33.3	58.3	8.3	32.0	44.0	24.0	94.7	5.3	0	37.5	37.5	25.0
Suicide		84.7	13.9	1.4	75.7	18.9	5.4	62.5	37.5	0	44.0	48.0	8.0	73.7	15.8	10.5	56.3	31.3	12.5
Unauthorized Leave		63.9	26.4	9.7	54.1	32.4	13.5	62.5	33.3	4.2	56.0	32.0	12.0	68.4	31.6	0	68.8	25.0	6.3
Substance Abuse		48.6	41.7	9.7	59.5	29.7	10.8	70.8	25.0	4.2	80.0	16.0	4.0	78.9	10.5	10.5	62.5	18.8	18.8
Self-Neglect		59.7	29.2	11.1	45.9	40.5	13.5	62.5	29.2	8.3	76.0	16.0	8.0	68.4	31.6	0	56.3	25.0	18.8
Victimization		61.1	29.2	9.7	43.2	43.2	13.5	62.5	37.5	0	68.0	24.0	8.0	89.5	10.5	0	66.7	20.0	13.3

### Lipid profile: diagnosis and sex effects

The mean TC level for the whole sample was 4.57 mmol/l (s.d. = 1.09). Within the sample, there was a significant main effect of diagnosis [*F* (2,223) = 5.04, *p* = 0.007; *np*^2^ = 0.043], with lower TC in both PD and DD groups, compared to the SZ group (PD vs SZ comparison, *p* = 0.028; DD vs SZ comparison, *p* = 0.004) (Table 1). PD and DD groups had comparable TC levels. There was no sex effect [*F* (1,223) = 0.04, *p* = 0.85; *np*^2^ = 0.0], and no diagnosis × sex interaction [*F* (2,223) = 1.01, *p* = 0.37; *np*^2^ = 0.009]. The use of statins was more prevalent in the PD (54.5%) relative to the SZ and DD groups (<9%), but TC levels in patients with or without statin use were similar in all three groups (Table 1).

For HDL, there was only a main effect of sex [*F* (2, 200) = 7.01, *p* = 0.009; *np*^2^ = 0.034], with males showing lower values than females [mean mmol/l (s.d.): males, 1.17 (0.49) mmol/l; females, 1.38 (0.56)]. There was no effect of diagnosis [*F* (2, 200) =0.11, *p* = 0.89; *np*^2^ = 0.001; mean mmol/l (s.d.): SZ, 1.24 (0.51); PD, 1.25 (0.55); DD, 1.31 (0.57] and no diagnosis x sex interaction [*F* (2, 200) =0.34, *p* = 0.71; *np*^2^ = 0.003] (Table 1).

For TG, there was only a main effect of diagnosis [*F* (2,187) = 4.47, *p* = 0.01; *np*^2^ = 0.046], with lower TG in the PD, relative to the SZ group (*p* = 0.004), and no significant difference between the PD and DD groups, or the DD and SZ groups [mean mmol/l (s.d.): SZ: 2.36 (1.31); PD: 1.88 (0.93); DD: 2.25 (0.83)] (Table 1).

### Lipid profile: association with risk of violence to others

For mean TC, there were significant inverse correlations with the number of days in seclusion due to high levels of violence, and the total number of days on high levels of observation due to violence, be it 1:1 or 2:1 (Table 3). These inverse associations were present with similar (i.e., small) effect sizes in all three groups and both sexes. No such correlation was found with days in long-term segregation due to violence. For TG and HDL, no significant correlations were found (Table 3).

**Table 3.** Correlations between lipids and measures of violence across the entire sample

Violence risk indicators	Correlation	TC (*n* = 230)	HDL (*n* = 207)	TG (*n* = 194)
Days due to violence	*r* [CI, 95%]	−0.088 [−0.215 to 0.042]	−0.059 [−0.194 to 0.078]	−0.024 [−0.164 to 0.117]
	*p*	0.18	0.40	0.74
Days 2:1	*r* [CI, 95%]	−0.129 [−0.254 to 0.000]	−0.028 [−0.108 to 0.164]	−0.063 [−0.202 to 0.078]
	*p*	**0.05**	0.68	0.38
Days due to violence seclusion	*r* [CI, 95%]	−0.132 [−0.257 to –0.003]	−0.055 [−0.190 to 0.082]	−0.026 [−0.166 to 0.116]
	*p*	**0.045**	0.43	0.72
Days on high levels due to violence	*r* [CI, 95%]	−0.180 [−0.313 to 0.039]	−0.024 [−0.171 to 0.124]	−0.144 [−0.293 to 0.012]
	*p*	**0.012**	0.75	0.07

When exploring the information from the structured professional judgment tools HCR-20 and START in relation to lipids, there were no significant correlations found across the entire sample, in separate diagnostic groups, or males or females (all *p* > 0.10) except inverse correlations between TC and total H score (sum of the scores from all the historical items in the HCR-20) and suicide (START) in females, and between TC and affective instability (C4) of the clinical risk factors in the DD group (Table 4).

**Table 4.** Correlations between TC and measures of HCR-20 and START

Risk Measure	Correlation	Entire Sample	SZ	PD	DD	All Males	All Females
HCR–20 Total H	*rho* [CI, 95%]	−0.061 [−0.204 to 0.084]	0.022 [−0.181 to 0.223]	−0.055 [−0.312 to 0.209]	0.124 [−0.223 to 0.443]	0.058 [−0.215 to 0.042]	−0.244 [−0.441 to 0.025]
	*p*	0.40	0.83	0.68	0.47	0.55	**0.02**
	*n*	196	100	60	36	112	84
HCR–20 C4 Instability	*rho* [CI, 95%]	0.001 [−0.114― 0.145]	0.050 [−0.153 to 0.250]	0.079 [−0.186 to 0.333]	−0.450 [−0.684 to 0.133]	−0.027 [−0.217 to 0.165]	0.019 [−0.202 to 0.239]
	*p*	0.99	0.62	0.55	**0.006**	0.78	0.86
	*n*	196	100	60	36	112	84
START Suicide	*rho* [CI, 95%]	−0.083 [−0.221 to 0.058]	−0.125 [−0.310 to 0.068]	0.130 [−0.131 to 0.374]	−0.228 [−0.533 to 0.130]	−0.019 [−0.163 to 0.200]	−0.214 [−0.416 to 0.008]
	*p*	0.23	0.19	0.31	0.19	0.83	**0.05**
	*n*	207	111	62	34	124	83

### Lipid profile: association with risk of self-harm and suicidality

There was no significant correlation between low TC levels and suicidality (START items) when examined across the entire sample, in separate diagnostic groups, or in males (all *p* > 0.10) but there was a marginal association in females (*p* = 0.05; Table 4).

## Discussion

Addressing the first study aim, the findings indicated lower TC in our UK forensic psychiatric sample, relative to recently reported TC norms for the UK population [67]. A World Health Organization (WHO) study involving several countries [67] reported the age-and sex-adjusted mean TC level in the UK to be 4.91 mmol/l [specifically for males, age 30–39 years, 5.02 mmol/l (95% CI: 4.98–5.05), and for females of the same age, 4.81 mmol/l (4.78–4.84)]. We found the mean TC levels in our SZ (4.77 mmol/l), PD (4.11 mmol/l), and DD (4.23 mmol/l) groups to be generally lower than these UK norms [67] with no significant effect of Sex and no diagnosis × sex interaction. Importantly, PD or DD patients had even lower TC than those with SZ. A previous study conducted in Finland [61] also found TC levels in male forensic inpatients to be somewhat lower (5.4 mmol/l) than the population norm (5.6 mmol/l). Taken together these findings suggest that TC levels for patients being admitted to forensic inpatient services may be lower than the national norms, and this may need to be borne in mind when considering cholesterol-lowering interventions in these forensic populations to reduce their cardiovascular risk. Our finding of higher HDL in females, compared to males, regardless of diagnosis is in line with sex differences (higher in females than males) in HDL seen in the general population [68].

In relation to the second aim, we found a small but significant link between low TC and violent behavior towards others across the three diagnostic groups and sexes. Our findings demonstrated such a link not only for SZ, where the evidence for such a link was previously suggested [49], but also for PD and DD, and that it also exists for female patients. In this study, we considered multiple measures to index risk, including days under restrictive interventions like seclusion and close observations as well as risk assessment tools such as the HCR-20, which was suggestive of a similar link. Specifically, the negative correlation between TC levels and days spent in seclusion due to violence, as well as the number of days on high observation levels due to violence, suggest low TC levels in the most violent patients in forensic settings, as they will be subject to these more restrictive interventions. Though these findings are generally consistent with previous research in psychosis [49] and PD [61, 62], ours is the first study to show such a link for women with psychosis in forensic settings and represents the largest study to demonstrate such an association in PD. The present study is also the first to report such a link in DD forensic inpatients. Overall, there is likely to be a significant though small association between lower-than-normal TC and risk of violence towards others in forensic inpatients which may be evident in studies with sufficient power and have pragmatic value for risk prediction when combined with other predictors.

Our study, however, did not demonstrate a clear link between TC levels and self-harm or suicidality in males, with only a marginally significant association found in females. This could be explained by the relatively low number of suicide attempts and that the patients were being actively treated with antidepressants and mood stabilizers. Misiak et al. [69] also did not find an association between TC and suicidal ideation and suggested that the mechanism behind lipid metabolism in suicidal patients might be more complex than previously thought. Other recent studies [70, 71] have also not found such a link. Taken together, these findings suggest a mechanism of action that may be, at least partially, different from the one for violence when exploring the link between lipids and suicidality. Lastly, no significant associations of HDL or TG were found with the risk of violence or suicidality in our study. Eriksen et al. [50] reported low HDL levels to be associated with violence in men, but not women, and hypothesized that HDL may be associated with different types of aggression, with women being more likely to show suicidal aggression. In our sample, as discussed earlier, there was a relatively low number of suicide attempts, and thus limited power to examine this possibility meaningfully.

With regard to the potential mechanism of an association between TC and risk of violence, cholesterol is a lipid that realizes many important functions in the cell: one of them is cell signaling, since cholesterol is essential for lipid rafts, structures necessary for multiple cellular functions [72]. Changes in cholesterol concentrations in the brain alter CNS functions and have been linked to neuroinflammation or serotonin hypofunction [71]. This contributes to reduced serotoninergic receptor activity, which leads to impulsivity and poor suppression of aggressive behavior [72, 73] contributing in turn to increased aggression and suicidal behavior [49]. Further work is needed to systematically investigate the impact of LDL, HDL and TG on objective measures of aggression, impulsivity and suicidal behavior in clinical and non-clinical populations.

Another observation deserving some comment is that we saw very low prescribing of statins in the SZ group, but this was more common in the PD group, possibly due to the PD group having more contact with mental health services prior to admission, which fits in with clinical experience. Consideration might have to be given to setting a different threshold for the prescription of statins for patients in secure care.

Limitations of the study include not being able to control for psychotropic drug treatment, with antipsychotic use frequently seen in the sample, especially in SZ and DD. Such drugs can interfere with lipid metabolism [74]. There were too many LDL results missing to conduct a meaningful analysis. Recording of information from risk assessment tools (HCR-20, START) was dependent on how these tools were filled out by the clinicians and data as recorded in Care Notes (and provided to the research team), without interviewing clinicians or patients to seek further clarity on missing or uncertain data. Our study lacked sufficient power to allow a meaningful examination of sex differences in lipids-violence associations. Based on the correlations we observed, 237 participants per group/subgroup would be required (providing 80% power at *p* < 0.05) to find the association between lower TC and “Days on high levels due to violence” (observed *r* = 0.18), and an even larger sample of 459 participants to find the associations between lower TC and ‘Days 2:1 observation due to violence’ or ‘Days due to violence seclusion’ (for both, observed *r* = 0.13), as determined using G*power3 [75].

## Conclusions

This study showed lower TC levels in patients admitted to forensic secure care regardless of specific diagnosis or sex. Our findings suggest that a low TC-violence link exists not only for men but women too, and not just for schizophrenia but also for PD and DD, and argue for a greater exploration of this link as a biological marker to aid risk prediction in forensic populations. Further work is needed to establish whether cholesterol levels are more a static marker of risk based on early life experiences, as opposed to a dynamic marker that alters with changes in risk levels. For such a study, it would be ideal to collect longitudinal data on lipid levels for the study sample, preferably starting early in at-risk samples (e.g., children with conduct disorder), along with a dynamic evaluation of their risk using multiple measures, including risk incidents as well as structured professional judgment tools like HCR-20 and START.

## Data Availability

All data supporting this work will be made freely available via Brunel University London research repository.
