# Low cholesterol and risk of violence in forensic inpatients with schizophrenia, personality disorder or dual diagnosis: same or different? – CORRIGENDUM

**DOI:** 10.1192/j.eurpsy.2025.10078

**Published:** 2025-09-23

**Authors:** Piyal Sen, Mehr-un-Nisa Waheed, Fern Taylor, Rebecca Mottram, Quazi Haque, Alexandra I. Blakemore, Veena Kumari

**Keywords:** lipids, risk assessment, schizophrenia, secure, statins, corrigendum

This article was originally published with a spelling error in the name of author Alexandra I. Blakemore. This has now been rectified and this corrigendum published.
